# Extracorporeal Membrane Oxygenation in Postcardiotomy Cardiogenic Shock

**DOI:** 10.14797/mdcvj.1256

**Published:** 2023-08-01

**Authors:** Bassel Akbik, Lin-Chiang P. Chou, Janardhana Gorthi

**Affiliations:** 1 Center for Critical Care, Houston Methodist, Houston, Texas, US; 2 Houston Methodist Cardiovascular Surgery Associates, Houston, Texas, US; 3 Houston Methodist DeBakey Cardiology Associates, Houston, Texas, US

**Keywords:** postcardiotomy shock, ECMO, cardiogenic shock, LV unloading

## Abstract

Postcardiotomy extracorporeal membrane oxygenation (PC-ECMO), the most frequent indication for ECMO in the United States, is increasingly used as the first-line mechanical circulatory support in patients who are refractory to conventional treatment. Despite increasing use of PC-ECMO, limited evidence is available regarding its safety, efficacy, and optimal timing for initiation and weaning. The decision to use PC-ECMO often is made in the absence of robust clinical data, leading to variability in patient selection, management, and outcomes across different institutions. This article summarizes current evidence on ECMO use in postcardiotomy cardiogenic shock and discusses its potential benefits, management, complications, and outcomes.

## Introduction

Cardiogenic shock following cardiac surgery is a life-threatening condition characterized by severe myocardial contractile impairment and reduced organ perfusion. Postcardiotomy cardiogenic shock (PCCS) occurs in 2% to 6% of patients undergoing surgical revascularization or valvular surgery.^1,2,3^ The incidence of refractory PCCS in adult cardiac surgical patients ranges from 0.5% to 1.5%.^4,5,6,7^ Without the use of mechanical circulatory support (MCS), PCCS is nearly always fatal.^1^

In 1966, Michael DeBakey used the first left ventricular assist device (LVAD) to support a patient who was in refractory PCCS. Following the first heart transplantation by Christiaan Barnard in 1967 and widespread acceptance of transplantation, MCS devices have been regularly used to sustain those patients in refractory PCCS to allow time for decisions regarding further management with implantable devices or heart transplantation.^1,8^

PC-ECMO has become the most frequent indication for ECMO in the United States (US).^7,9^ It is increasingly used as first-line MCS in patients who are refractory to conventional treatment. PC-ECMO facilitates respiratory gas exchange and provides cardiac output to end organs, supporting organ recovery and allowing time for “bridge-to-decision” to more durable modes of support.^7,9,10,11^

The published reports of PC-ECMO are mostly single-center experiences. Despite increasing use of PC-ECMO, there is limited evidence on its safety, efficacy, and optimal timing of initiation and weaning. The decision to use PC-ECMO is often made in the absence of robust clinical data, leading to variability in patient selection, management, and outcomes across different institutions. Moreover, PC-ECMO is associated with a high risk of complications, which can further impact patient outcomes and healthcare costs. In this article, we aim to summarize the current evidence on ECMO use in PCCS and discuss its potential benefits, management, complications, and outcomes.

## Characteristics of Patients Needing PC-ECMO

The nature of the PC-ECMO indication is that patients invariably require urgent or emergent ECMO support compared with other indications. Patients requiring PC-ECMO are usually older, with a higher incidence of renal insufficiency, prior myocardial infarction, left main coronary artery disease, and left ventricular (LV) dysfunction. They also are more likely to have a prolonged history of coronary artery disease and prior open-heart surgery.^9,12^

PC-ECMO is most frequently associated with patients who undergo coronary artery bypass grafting (CABG) (26.8%) and valve surgery (25.6%) followed by post-heart transplantation (20.7%). The use of ECMO in post-heart transplant recipients has been reported to be as high as 10% to 15% of patients.^9,13,14,15,16^

## Indications

The most common indication for PC-ECMO implementation is intraoperative failure to wean from cardiopulmonary bypass (CPB) because of univentricular, biventricular, or delayed refractory cardiogenic shock; postoperative cardiac arrest in the intensive care unit (ICU); respiratory failure; or intractable postoperative ventricular arrhythmias.

PC-ECMO is also used for early graft dysfunction in post-heart transplant recipients.^14,15,16^ Another indication for PC-ECMO is in patients who develop right ventricular failure after LVAD implantation. PC-ECMO may be utilized to support the right ventricle (RV) or to bridge to an RV assist device.^17,18^

The use of ECMO for PC cardiac arrest has been increasingly implemented during the last 10 years. The 2020 European Association for Cardio-Thoracic Surgery/Extracorporeal Life Support Organization/Society of Thoracic Surgeons/American Association for Thoracic Surgery Expert Consensus for the Resuscitation of Patients who arrest after Cardiac Surgery recommended that failure to achieve spontaneous circulation is an indication for open cardiac massage and the institution of either central or peripheral ECMO at the bedside.^19^

## Implementation and Management of PC-ECMO

Operating room (OR) cannulation, either centrally or peripherally, occurs most frequently, followed by the ICU and rarely on the ward. Most PC-ECMO placements occur within 24 to 48 hours of procedure, and patients are rarely supported on ECMO for more than 15 days.

The choice of ECMO cannulation site depends on several factors, including timing, indication, urgency of deployment, and the need for cardiocirculatory or respiratory support as well as institutional considerations such as staff familiarity and availability of ECMO circuits.

**Figure 1:**
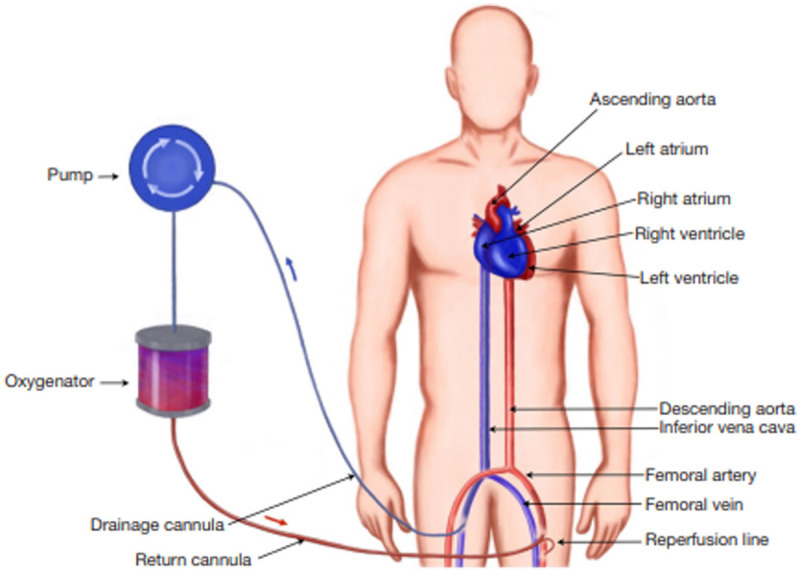
Peripheral extracorporeal membrane oxygenation cannulation approach.

When opting for central PC-ECMO cannulation, an open sternum is typically required to accommodate the atrial and aortic cannulas. This approach provides improved cardiac decompression and anterograde flow from the proximal aorta compared to peripheral cannulation, thereby mitigating the potential complications associated with retrograde flow, such as “Harlequin syndrome.” However, it is worth noting that central cannulation is associated with higher rates of bleeding and renal failure (Figure 1).^9^

**Figure 2:**
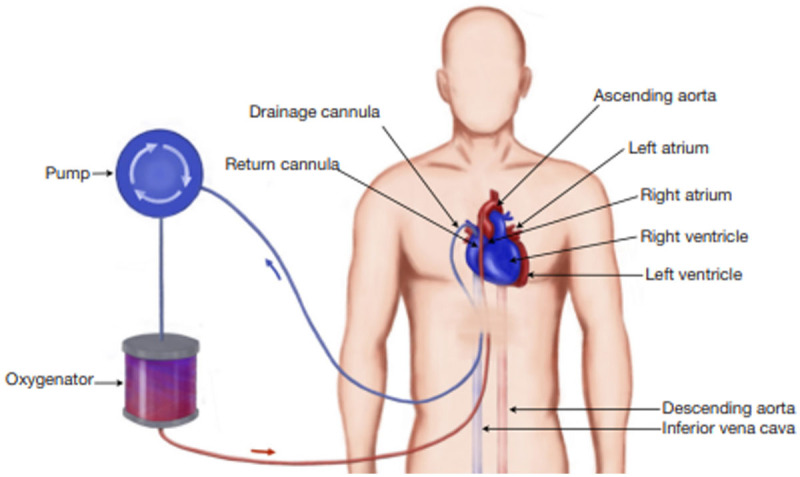
Subxiphoid tunneled cannulation approach.

Innovative strategies have been developed to address the management of atrial and aortic cannulas in central cannulation. These strategies involve sternal closure with tunneled cannula exit either in the subxiphoid or suprasternal region. Such approaches offer several benefits, including facilitating patient extubation and mobilization, while reducing the risk of infection (Figure 2).^9^

Venoarterial ECMO (VA-ECMO) is effective in draining the right side of the heart but may not adequately decompress the LV due to incomplete drainage of systemic and bronchopulmonary venous flow. This can lead to LV distension caused by impaired LV contractility or high afterload, resulting in inadequate ejection. In turn, insufficient LV ejection can cause blood stasis in the left cardiac chambers, increasing the risk of clot formation and catastrophic consequences. To mitigate these risks, the use of MCS devices for LV unloading is increasingly considered. Clinical studies and literature analyses suggest improved outcomes when adjunctive LV unloading strategies are employed.^20^

Various indirect and direct modalities for LV unloading have been described, including intra-aortic balloon counterpulsation pump (IABP), Impella, atrial transseptal catheterization, atrial septostomy, pulmonary artery drainage/venting, transaortic venting, and direct left atrial and LV venting.^20^

The use of IABP in conjunction with VA-ECMO is a topic of debate. When combined with VA-ECMO, IABP is employed to improve coronary perfusion and support LV ejection and blood flow pulsatility by reducing afterload and LV wall tension, thereby decreasing blood stasis in the left heart. The concomitant use of IABP with PC-ECMO does not appear to increase the risk of limb ischemia.^9,16,21,22,23^

Several clinical studies have investigated the role of Impella for unloading/venting in VA-ECMO-treated patients with cardiogenic shock. Pappalardo et al. described a large series of patients treated with the combination of VA-ECMO and Impella compared to patients treated with VA-ECMO alone. Despite a higher need for continuous renal replacement therapy (48% vs 19%) and increased hemolysis (76% vs 33%), the combination group had significantly lower hospital mortality (47% vs 80%) and a higher rate of successful bridging to recovery or further therapy (68% vs 28%) than the VA-ECMO alone group.^20,24,25,26,27^

In another international multicenter cohort study involving 686 patients with cardiogenic shock treated with VA-ECMO, the use of LV unloading with Impella during VA-ECMO was associated with lower mortality compared with VA-ECMO support alone (58.3% vs 65.7%).^28^

## Major Complications with PC-ECMO

The use of PC-ECMO is associated with numerous complications, although the number of patients with major complications has decreased in recent years.^13^ Major hemorrhage is the most reported complication after the institution of VA-ECMO. It has led to reintervention in almost half of the patients in some studies.^5,29,30,31,32,33^ Many investigators advocate avoiding heparin infusion for the first 12 to 48 hours provided that high flows are maintained to prevent clot formation.^34^

Central nervous system complications have recently been shown to occur in 15% of adult patients supported with VA-ECMO and 7% of adult patients supported with VV-ECMO.

In PC-ECMO, these rates may be much higher, occurring in up to 30% of patients.^35,36,37,38^

In a single-center retrospective study of 496 PC-ECMOs, neurologic complications occurred in 87 patients (21.0%), including cerebral infarction in 33 (8.0%), brain death in 30 (7.2%), seizures in 14 (3.4%), and intracranial hemorrhage in 11 (2.7%). In-hospital mortality in patients with neurologic complications was 90.8% compared with 52.1% in control patients. Low systolic blood pressure level pre-ECMO and aortic surgery combined with coronary artery bypass grafting were associated with high neurologic complications.^34^

Renal failure requiring renal replacement therapy was another commonly reported complication. The incidence of renal failure requiring renal replacement therapy while on PC-ECMO had a strong association with mortality.^5,29,30,33,39^

With regard to limb ischemia in peripherally cannulated VA-ECMO, several investigators have consistently reported reduced limb ischemia with the routine use of ipsilateral distal perfusion. A delay in distal perfusion cannula placement, triggered by signs of ischemia, may lead to a reperfusion injury with a poor outcome.^35^

## Weaning Protocol

Weaning off ECMO requires a thorough assessment of various parameters. Echocardiographic indices are commonly used to evaluate readiness to be weaned, along with other parameters such as biomarkers or hemodynamic parameters. Signs of persistent organ damage are negatively associated with weaning outcomes. Higher aspartate aminotransferase levels at 48 and 72 hours after ECMO initiation are linked to weaning failure.^35,40,41^

Pulse pressure (PP) less than 30 mm Hg is independently associated with weaning failure.^35,42^ In contrast, successfully weaned patients exhibit favorable right ventricular hemodynamic parameters at 48 and 72 hours, including lower right atrial (RA) pressure to pulmonary capillary wedge pressure ratio, lower transpulmonary gradient, and higher pulmonary artery pulsatility index.^35,43^

The early recovery of LVEF after VA-ECMO initiation is linked to improved weaning outcomes. The most widely used parameter to track LV recovery in patients on VA-ECMO is the LV outflow tract velocity time integral (VTI), with a threshold of greater than 9.5 cm commonly reported to predict successful weaning.^35,44,45,46,47,48,49,50,51^ Additionally, improvements in lateral e’ velocity and tricuspid annular S’ velocity during a flow reduction trial are predictors of weaning success.^35^

Ventricular interdependence absence is also a robust predictor of successful weaning, with a sensitivity of 94% and specificity of 95%.^52^

Suggested criteria for weaning from VA-ECMO include maintaining a mean arterial pressure equal or greater than 60 mm Hg with minimal inotropic and vasopressor support, lactate normalization, SvO_2_ greater than 65%, absence of ventricular arrhythmia, optimized fluid balance, adequate native lung oxygenation capacity with resolution of pulmonary edema, and an inspired oxygen fraction less than 50%. Echocardiographic improvement in myocardial function is evident by S’ > 6 cm/s, left ventricular ejection time > 200 ms, LV outflow tract VTI = 10 cm, and tricuspid annular plane systolic excursion > 10 mm.^35^

Once these criteria are met, a gradual reduction of ECMO blood flow to 0.5-1 L/min (maintaining a minimum rotational speed of 1,500 RPM) with careful monitoring of hemodynamics, vasopressor needs, and biventricular response to load variation can be initiated. When this is achieved, the arterial and venous lines can be clamped for 1 to 2 min (on therapeutic anticoagulation). If the LV ejection fraction was >35% to 40%, VTI was > 10 cm, and cardiac index (CI) was > 2.2 L/min/m^2^, then removal of ECMO can be considered with surgical repair of the mediastinum or the peripheral vascular access. Some centers recommend maintaining EC blood flow at between 1.5 and 2 L/min for 24 h with hemodynamic stability prior to ECMO removal.

The use of pump-controlled retrograde trial off (PCRTO) instead of circuit clamping has been reported,^35^ which reduces the risk of circuit thrombosis. During PCRTO, pump speed is gradually reduced until the flow becomes retrograde, creating a controlled arteriovenous fistula. Retrograde flow is maintained for up to 1 hour, during which time the sweep gas is turned off to assess any residual gas exchange impairment. If the patient remains stable, and hemodynamic parameters, vasopressor needs, and oxygen requirements remain within acceptable ranges, the trial is considered successful. It is important to note that reversing the flow during PCRTO comes with a theoretical risk of pulmonary embolism through clot detachment from the venous side of the oxygenator. Further studies are necessary to validate the safety and efficacy of this weaning method.^35,53^

## PC-ECMO Outcomes

Analysis of the Extracorporeal Life Support Organization (ELSO) registry for patients on PC-ECMO from January 2010 through December 2018 showed that successful weaning from ECMO was possible in 56.4% of patients, while survival to hospital discharge was achieved in 41.7% of cases. Higher mortality was observed after CABG (65.4%), combined CABG with valve (68.4%), and aortic procedures (69.6%) when compared with other indications. Lower mortality rates were observed in heart transplantation recipients (46.0%).^13^

Pozzi et al. described similar outcomes in 90 patients who required PC-ECMO between January 2007 and December 2017 in France; in this report, successful weaning was accomplished in 50% of patients after a mean support 6.4 days, and 39% of patients survived to hospital discharge.^54^

Older age is significantly associated with hospital death. Patients over 70 years of age had lower survival rate to discharge compared with those ≤ 70 years old.^13^

In several experiences, extracorporeal cardiopulmonary resuscitation (ECPR) is a strong negative predictor of survival.^9,55,56^ A trend toward lower in-hospital mortality was reported with peripheral VA-ECMO cannulation compared with central cannulation.^13^

The timing of PC-ECMO implementation before organ hypoperfusion progresses is very important. Fux and collaborators have shown that a lactate value above 10 mmol/L at implant is associated with 90% in-hospital mortality, with no survival when lactate levels were > 15 mmol/L.^7^ Another study found that a pre-ECMO implantation lactate level of greater than 6.45 mmol/L was associated with higher mortality. Patients who showed decreasing lactate after PC-ECMO support had higher success rates of weaning off ECMO.

## Conclusion

ECMO has been increasingly used to support patients with refractory PCCS, which is invariably a fatal clinical state. Despite advancements in ICU management and ECMO circuit components and technologies, mortality rates have not significantly decreased over time. Further improvement in patient selection, early implantation of ECMO before tissue hypoperfusion, LV unloading, cannulation approach, anticoagulation protocols, and ECMO management are important to improve these patients’ outcomes.
